# Editorial: Bioinformatics and systems biology strategies in disease management with a special emphasis on cancer, Alzheimer’s disease and aging

**DOI:** 10.3389/fmolb.2025.1725000

**Published:** 2025-10-31

**Authors:** Tiratha Raj Singh, Brigitte Vannier, Ragini Raj Singh, Hari Om Yadav, Holger Fröhlich

**Affiliations:** ^1^ Department of Biotechnology and Bioinformatics, Centre of Healthcare Technologies and Informatics (CEHTI), Jaypee University of Information Technology (JUIT), Solan, Himachal Pradesh, India; ^2^ CoMeT Laboratory, Université de Poitiers, Poitiers, France; ^3^ Department of Physics and Materials Science, Jaypee University of Information Technology (JUIT), Solan, Himachal Pradesh, India; ^4^ Department of Neurosurgery and Brain Repair, Morsani College of Medicine, Centre for Microbiome Research, Microbiomes Institute, Byrd Alzheimer’s Centre and Research Institute, University of South Florida, Tampa, FL, United States; ^5^ Bonn-Aachen International Centre for Information Technology (b-it), University of Bonn, and Fraunhofer Institute for Algorithms and Scientific Computing (SCAI), Sankt Augustin, Germany

**Keywords:** bioinformatics, systems biology, cancer, alzheimer’ disease, omics, disease management, aging

## Abstract

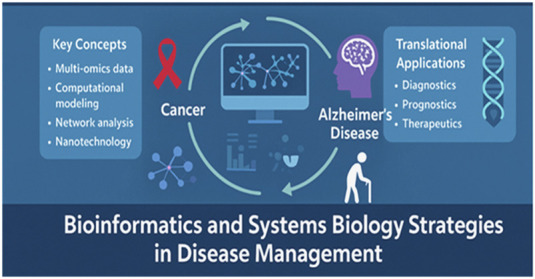

## Introduction

Advances in bioinformatics and systems biology are transforming how we understand and manage complex diseases by integrating multi-omics data, computational modeling, network analysis, and nanotechnology. Cancer, Alzheimer’s disease (AD), and the biological processes underlying aging remain among the most pressing global health challenges. Despite their differences, these conditions share a unifying theme: they are multifactorial, dynamic, and deeply interconnected with molecular networks that cannot be fully captured by single-gene or reductionist perspectives. The goal of this Research Topic is to highlight how computational approaches, data-driven models, and systems-level thinking are reshaping strategies for disease management. By bringing together contributions that span computational biology, network medicine, biomarker discovery, and translational modeling, this Research Topic emphasizes not only the depth of current knowledge but also the integrative frameworks necessary to drive future innovation.

## Aims and rationale

The overarching goal of this Research Topic is to spotlight cutting-edge research at the interface of bioinformatics, systems biology, and translational medicine, focusing on cancer, Alzheimer’s disease (AD), and aging. By fostering interdisciplinary collaboration among specialists in biology, medicine, computer science, engineering, and nanotechnology, the Research Topic seeks to accelerate the translation of computational and experimental insights into tangible clinical outcomes.

## Summary of contributions

### Alzheimer’s disease and dementia

- *Artificial intelligence and omics-based autoantibody profiling in dementia* employs AI to dissect autoantibody signatures, offering insights into neurodegenerative immunological patterns Matsuda et al.
- *Development of a Novel Diagnostic Model for Alzheimer’s Disease* leverages glymphatic system–and metabolism-related gene expression to build a predictive model for AD diagnosis Jiang et al.
- *Reorganized brain functional network topology in stable and progressive mild cognitive impairment* revealed significant differences in network topological properties among sMCI, pMCI and HC patients, which were significantly correlated with cognitive function Xue et al. Most notably, the cerebellar module played a crucial role in the overall network interactions.

### Cancer

- *Immunological biomarkers and gene signatures predictive of radiotherapy resistance in NSCLC* identify key immune-related markers that may forecast treatment response and inform precision oncology Lv et al.
- *Identification of kidney renal clear cell carcinoma prognosis based on gene expression and clinical information* presents a prognostic modeling framework integrating genomics and clinical data, with potential implications for patient stratification and personalized therapy Zou et al.
- *Computational molecular insights into ibrutinib as a potent inhibitor of HER2-L755S mutant in breast cancer* utilizes virtual screening, docking, gene expression profiles, and molecular dynamics to elucidate the molecular underpinnings of ibrutinib’s therapeutic potential Loganathan and Doss.
- *Elucidating the multiscale mechanisms and therapeutic targets of caffeic acid in gastric cancer: A synergy of computational and experimental approaches.* This study confirmed that caffeic acid regulates FZD2 expression and inhibits the activation of the noncanonical Wnt5a/Ca^2+^/NFAT signaling pathway, thereby interfering with gastric cancer–related pathological processes Zhang et al. These findings reveal the molecular mechanism of caffeic acid in gastric cancer and reflect the value of natural products in cancer research.
- *A comprehensive analysis of the prognostic value, expression characteristics and immune correlation of MKI67 in cancers.* This study aims to perform a comprehensive pan-cancer analysis of the prognosis value of Ki67 across various cancer types Pan et al. Nuclear-associated antigen Ki67 (Ki67) emerges as a clinically practical biomarker for proliferation assessment among many cancer types.

### Cross-domain themes

Although not directly represented among the submitted articles, this Research Topic’s conceptual foundation highlights multi-omics integration, network theory, and systems-level drug discovery. Approaches like signaling network entropy and network-based drug repositioning reinforce the thematic unity of this Research Topic.

## Translational opportunities and challenges

While the potential of bioinformatics and systems biology is immense, several challenges remain. Translational integration into clinical workflows requires overcoming issues of data standardization, reproducibility, and interpretability. Despite these challenges, the contributions in this Research Topic point toward a future where computational and systems approaches guide every stage of disease management from early detection and patient stratification to therapeutic design and monitoring. By bridging fundamental biology with clinical application, bioinformatics and systems biology provide a roadmap toward personalized, predictive, and preventive medicine.

## Context and outlook

This Research Topic reflects the broader trajectory of biomedical science: moving beyond reductionist models toward holistic, systems-level frameworks. Multi-omics and systems biology bridge the gap between genotype and phenotype, revealing dynamic networks and regulatory architectures that drive disease. With the emergence of AI, machine learning, and experimental validation pipelines, we now have the tools to translate systems insights into diagnostics, prognostics, and therapeutics especially for multifactorial conditions like cancer and AD.

Looking ahead, the integration of nanotechnological delivery systems, *in silico* modelling, and cross-modal data fusion (e.g., imaging-omics, longitudinal cohorts) will further accelerate the path to precision medicine, ageing interventions, and personalised disease management.

## Conclusion

This Research Topic illustrates how the synergistic use of bioinformatics and systems biology is reshaping our understanding and management of complex diseases, with cancer, Alzheimer’s disease, and aging serving as paradigmatic examples. The contributions highlight not only the scientific advances but also the translational potential of integrative strategies that embrace complexity rather than reduce it.

As the biomedical community moves forward, the challenge will be to harness these insights in ways that are equitable, clinically actionable, and globally relevant. We hope this Research Topic stimulates further dialogue and research, inspiring multidisciplinary collaborations that leverage data, models, and systems thinking to confront the pressing health challenges of our time.

